# Retraction Note: Factors affecting awareness of emergency contraception among college students in Kathmandu, Nepal

**DOI:** 10.1186/s12905-023-02201-w

**Published:** 2023-02-09

**Authors:** Ramesh Adhikari

**Affiliations:** 1 Geography and Population Department, Mahendra Ratna Campus, Tribhuvan University, Kathmandu, Nepal; 2 Institute for Population and Social Research, Mahidol University, Salaya, Thailand

**Retraction Note: BMC Women‘s Health 2009, 27:9** https://doi.org/10.1186/1472-6874-9-27

The Editor has retracted this article due to the author’s inability to provide documentation of approval from an ethics committee.

The author does not agree to this retraction.

